# State of Charge Estimation of Li-Ion Battery Based on Adaptive Sliding Mode Observer

**DOI:** 10.3390/s22197678

**Published:** 2022-10-10

**Authors:** Qi Wang, Jiayi Jiang, Tian Gao, Shurui Ren

**Affiliations:** 1 School of Electronic Information Engineering, Xi’an Technological University, Xi’an 710032, China; 2 School of Electronic and Information, Northwestern Polytechnical University, Xi’an 710072, China

**Keywords:** Li-ion battery, state of charge, adaptive sliding mode observer, DP equivalent circuit

## Abstract

As the main power source of new energy electric vehicles, the accurate estimation of State of Charge (SOC) of Li-ion batteries is of great significance for accurately estimating the vehicle’s driving range, prolonging the battery life, and ensuring the maximum efficiency of the whole battery pack. In this paper, the ternary Li-ion battery is taken as the research object, and the Dual Polarization (DP) equivalent circuit model with temperature-varying parameters is established. The parameters of the Li-ion battery model at ambient temperature are identified by the forgetting factor least square method. Based on the state space equation of power battery SOC, an adaptive Sliding Mode Observer is used to study the estimation of the State of Charge of the power battery. The SOC estimation results are fully verified at low temperature (0 °C), normal temperature (25 °C), and high temperature (50 °C). The simulation results of the Urban Dynamometer Driving Schedule (UDDS) show that the SOC error estimated at low temperature and high temperature is within 2%, and the SOC error estimated at normal temperature is less than 1%, The algorithm has the advantages of accurate estimation, fast convergence, and strong robustness.

## 1. Introduction

Li-ion batteries have gradually become the main power source of new energy electric vehicles due to their high energy density, long cycle life, low self-discharge rate [1], and good safety [2], and determine the cruising range of the vehicle. State of Charge (SOC) characterizes the remaining battery capacity, which is the core content of Battery Management Systems (BMSs), and an important indicator to assess the current status of batteries, high-precision SOC estimation is a must for power battery pack control strategies [3,4]. However, the SOC as a state quantity cannot be measured directly [5] and is affected by many factors. Therefore, it must be estimated approximately by measuring some other physical quantities such as voltage, current, etc. [6], and using a mathematical model or algorithm [7]. Accurate estimation of SOC is an important prerequisite for multiple battery control strategies [8]. It is important for accurately estimating vehicle mileage, prolonging battery life, preventing single batteries from overcharging or overloading, ensuring the maximum efficiency of the entire battery pack [9], and improving the economy of batteries [10].

Currently, the main methods of battery SOC estimation include the current integration method [11], open-circuit voltage method [12], machine learning algorithm [13], Kalman Filter algorithm [14], and so on. The estimation accuracy of the current integration method depends greatly on the sampling frequency and the accuracy of the current sensor instead of hardware. The open-circuit voltage method requires a long period of static time (up to several hours) to ensure that the port voltage of the battery is exactly the open-circuit voltage of the battery [15]. It is difficult to apply to real-time estimation, and the estimation method is open-loop estimation, which has a low accuracy. The machine learning algorithms include Artificial Neural Network (ANN) [16], Fuzzy Logic Control (FLC) [17], Support Vector Machine (SVM) [18], etc. This algorithm requires a lot of data to be trained. This process is time-consuming and powerful, and it is only effective for training data. Document [19] selects the recursive least squares method with different forgetting factors to identify equivalent model circuit parameters, and uses the linear Kalman filter to estimate SOC. However, the SOC estimation algorithm based on the Kalman filter requires accurate battery model parameters [20], because the covariance of process noise and measurement noise is well known [21]. Due to the complex electrochemical reaction inside the battery under the driving condition of electric vehicles, this assumption is unrealistic and prone to errors. The working conditions of electric vehicles in the driving process are very complex, which will lead to chemical reactions in the battery that cannot be directly expressed by physical quantities, and unpredictable noise. Both of these will have a certain impact on the algorithm and lead to obvious errors. In addition, the constant value of noise covariance may cause significant error and divergence of SOC estimation results.

The Sliding Mode Observer (SMO) [22] is a kind of nonlinear state observer, which changes the control loop of the general state observer into a sliding mode variable structure form, making the system reach a stable state of small amplitude and high frequency. Zhou Juan [23] proposed a Sliding Mode Observer algorithm based on the joint extended Kalman filter, which was used to design SOC estimation experiments under variable temperature environments. The results showed that the maximum SOC estimation error is 3.55%. A method of SOC estimation with Sliding Mode Observer proposed by Khan Zeeshan Ahmad [24] has a convergence of 330 s at a normal temperature (25 °C). Based on the above research, aiming at the demand for high accuracy and fast convergence of SOC estimation, a SOC estimation method based on adaptive Sliding Mode Observer is proposed and designed in this paper, which can overcome the nonlinearity, external interference, and measurement noise of the battery model. This method has higher estimation accuracy with less than 2% and faster convergence.

This paper is organized into various sections where Section 1 is an introduction. In the second section, based on the establishment of the second-order equivalent circuit model with variable parameters at different temperatures, the least square method with a forgetting factor is used to identify the model parameters. This section also verifies the accuracy of the model under UDDS conditions. In the third section, according to the fully adaptive disturbance characteristics of sliding mode control, an adaptive Sliding Mode Observer is proposed and designed to estimate the SOC of the lithium-ion battery in electric vehicles. The fourth section carries out experimental verification at low temperature (0 °C), normal temperature (25 °C), and high temperature (50 °C) under constant current conditions and UDDS working conditions. Finally, a conclusion is presented on the estimation accuracy and convergence speed of the SOC estimation method based on the adaptive Sliding Mode Observer in the fifth section.

## 2. Establishment of an Equivalent Circuit Model for Li-Ion Batteries

In the process of establishing the equivalent circuit model of the battery, according to the OCV (Open Circuit Voltage)–SOC curve obtained from Hybrid Pulse Power Characteristic (HPPC) experiments, a DP equivalent circuit model is established. The parameters of ohmic internal resistance, the electrochemical polarization resistance, the concentration polarization resistance, the electrochemical polarization capacitance, and the concentration polarization capacitance are identified using the least squares with the forgetting factor method. The model is verified with the experimental data from the HPPC experiments, in order to ensure the accuracy and practicability of the model.

### 2.1. Second-Order Equivalent Circuit Model of Battery

**Figure 1:**
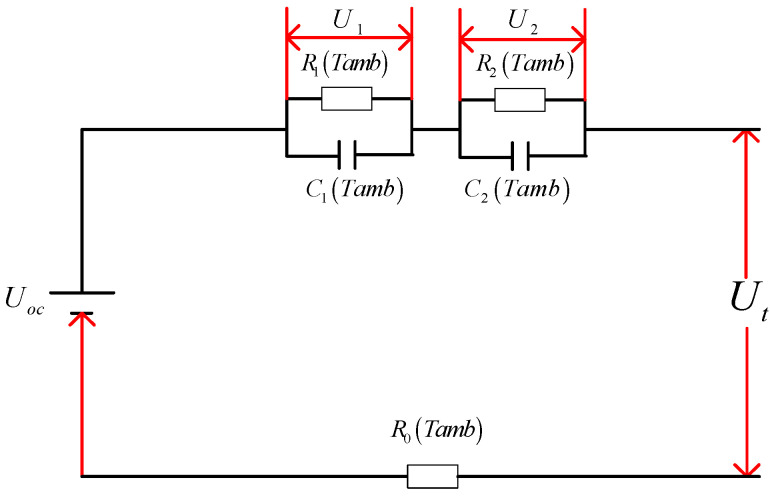
DP equivalent circuit diagram.

The second-order RC equivalent circuit model can accurately describe the dynamic [25] and static characteristics [26] of the battery, with low complexity [27,28] and easy engineering implementation [29,30]. Considering the influence of different ambient temperatures on the SOC of batteries, an improved model of parameters changing with temperature, the DP equivalent circuit model with temperature changing parameters, is established. The DP equivalent circuit model is shown in Figure 1.

In Figure 1, U_OC_ is the OCV and R_0_ (Tamb) is the ohmic internal resistance; R_1_ (Tamb) and R_2_ (Tamb) are the electrochemical polarization resistance and the concentration polarization resistance, respectively; C_1_ (Tamb) and C_2_ (Tamb) are the electrochemical polarization capacitance and the concentration polarization capacitance, respectively. According to Kirchhoff’s Law, the calculation formula for the terminal voltage of the DP equivalent circuit model is:

(1) $$\left\{\begin{array}{c} {\dot{U}}_{1}=-\frac{U_{1}}{R_{1}\left(Tamb\right)C_{1}\left(Tamb\right)}+\frac{I}{C_{1}\left(Tamb\right)}\\ {\dot{U}}_{2}=-\frac{U_{2}}{R_{2}\left(Tamb\right)C_{2}\left(Tamb\right)}+\frac{I}{C_{2}\left(Tamb\right)}\\ \begin{array}{l} \;U_{t}=U_{OC}-U_{1}-U_{2}-IR_{0}\\ \;{\dot{U}}_{OC}=-\frac{1}{C_{n}} \end{array} \end{array}\right.$$

### 2.2. Parameter Identification of the Battery Model

The 18,650 ternary lithium-ion battery with a nominal capacity of 2600 mAH and a nominal voltage of 3.7 V was used in the experiment. The HPPC discharge experiment method was used to obtain the relationship between the open circuit voltage of the battery and SOC. The basic method is to discharge 5% of the battery’s power every 1.5 h after the battery is fully charged. After the voltage is stabilized, the open circuit voltage corresponding to the current SOC is obtained. Finally, the corresponding data of OCV and SOC are fitted to obtain the relationship expression. The experiment is divided into the following six steps:

(a) Put seven batteries in the same healthy state in the incubator at −10, 0, 10, 25, 30, 40, and 50 °C for 2 h;
(b) Use the power battery performance test platform to discharge the single battery with 0.2 C current to the cut-off voltage of 2.5 V;
(c) After the battery is left for 2 h, charge the battery in the way of constant current first and then constant voltage according to the charging standard. When the battery is charged to 4.2 V, it is in the fully charged state by default, and the SOC is recorded as 1;
(d) Set the thermostat to −10, 0, 10, 25, 30, 40, and 50 °C, and let the battery stand for 2 h;
(e) In the constant temperature box, discharge the battery with 0.2 C current. After discharging to 5% of the standard capacity, let the battery stand for 2 h, and record the voltage at this time as the open circuit voltage;
(f) Repeat step (e) until the cut-off voltage is 2.5 V.

The IT 8500 discharge meter and UK-150G thermostat are used to monitor the discharge current and terminal voltage of the battery in real time.

#### 2.2.1. OCV Curve Fitting

**Figure 2:**
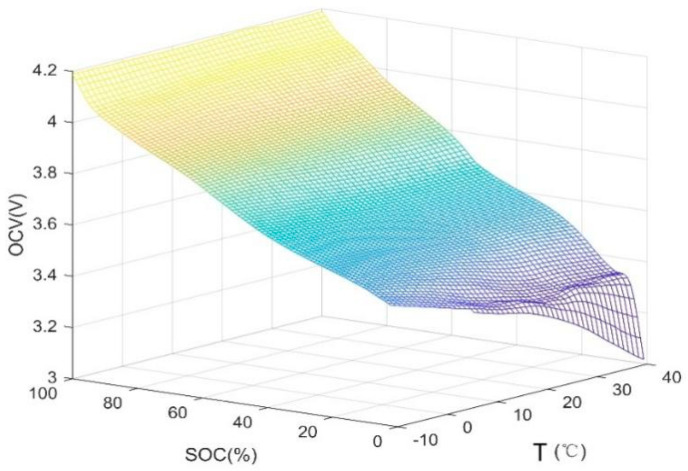
The OCV–SOC relationship curve at different temperatures.

At −10, 0, 10, 20, 25, 30, 40, and 50 °C, the fully charged battery was continuously discharged at 0.2 C between the equal interval points of SOC. For every 5% decrease in SOC, the battery open circuit voltage was recorded after standing for 1.5 h. The OCV–SOC relationship curve at different temperatures is shown in Figure 2.

As shown in Figure 2, through the above experimental process, the open-circuit voltage data collected at various temperatures are fitted to obtain a three-dimensional space model diagram. Figure 2 clearly shows the relationship between the three. It can be seen from the changes in the three parameters in the figure that under the same temperature, the larger the SOC value, the larger the open circuit voltage. Additionally, the increasing trend of open circuit voltage is more obvious, and its range is 3~4.2 V. When SOC < 20% and T > 10°C, at the same SOC, the open circuit voltage shows a decreasing trend with the increase in temperature, and the decreasing trend is obvious when the SOC approaches 0. When SOC > 20% and T < 10°C, the open circuit voltage changes at the same SOC. Therefore, to build a more accurate battery model, it is necessary to consider the effect of temperature on the open circuit voltage.

#### 2.2.2. Component Parameter Identification

Under the ambient temperature of [−10, 0, 10, 20, 25, 30, 40, 50], the Forgetting Factor Least Square (FFLS) is used to identify the parameters of the Li-ion battery model fused with ambient temperature. The calculation process of battery model parameter identification based on the forgetting factor least square method is as follows.

1. The system transfer function (2) is obtained by Laplace transform of the terminal voltage transformation Formula (1).

(2) $$G\left(s\right)=\frac{U_{OC}\left(s\right)-U_{t}\left(s\right)}{I\left(s\right)}=\frac{R_{0}s^{2}+\frac{1}{\tau_{1}\tau_{2}}\left(R_{0}\tau_{1}+R_{0}\tau_{2}+R_{1}\tau_{2}+R_{2}\tau_{1}\right)s+\frac{R_{0}+R_{1}+R_{2}}{\tau_{1}\tau_{2}}}{s^{2}+\frac{\left(\tau_{1}+\tau_{2}\right)}{\tau_{1}\tau_{2}}s+\frac{1}{\tau_{1}\tau_{2}}}$$

2. Discretization using bilinear transformation, let $s=\frac{2}{\Delta t}\frac{1-z^{-1}}{1+z^{-1}}$, then:

(3) $$G\left(z^{-1}\right)=\frac{a_{3}+a_{4}z^{-1}+a_{5}z^{-2}}{1-a_{1}z^{-1}-a_{2}z^{-2}}$$

Among them, $a_{i}\left(i=1,2,...,5\right)$ is a constant.

The difference equation is as follows:

(4) $$y\left(k\right)=a_{1}y\left(k-1\right)+a_{2}y\left(k-2\right)+a_{3}I\left(k\right)+a_{4}I\left(k-1\right)+a_{5}I\left(k-2\right)$$

Among them, $y\left(k\right)=U_{OC}\left(k\right)-U_{t}\left(k\right)$, $y\left(k\right)$ represents the pressure difference, and $I\left(k\right)$ is the input current.

1. Define

(5) $$\left\{\begin{array}{c} \varphi \left(k\right)={\left[y\left(k-1\right),y\left(k-2\right),I\left(k\right),I\left(k-1\right),I\left(k-2\right)\right]}^{T}\\ \theta =\left[a_{1},a_{2},a_{3},a_{4},a_{5}\right] \end{array}\right.$$

Define the sampling error as $e\left(k\right)$, then

(6) $$y\left(k\right)=\varphi^{T}\left(k\right)\theta +e\left(k\right)$$

2. Calculate the recursive termination condition $J\left(\theta \right)$.

From:

(7) $$\left\{\begin{array}{c} Y={\left[y\left(3\right),y\left(4\right),y\left(5\right)\ldots y\left(N+2\right)\right]}^{T}\\ e={\left[e\left(3\right),e\left(4\right),e\left(5\right)\ldots e\left(N+2\right)\right]}^{T} \end{array}\right.$$

Then:

(8) $$J\left(\theta \right)={\sum_{i=1}^{k}\left(Y-\phi \theta \right)}^{2}=\sum_{i=1}^{k}{\left(e\left(i+2\right)\right)}^{2}$$

1. Introducing the forgetting factor λ, the recursive formula of FFLS is as follows:

(9) $$\left\{\begin{array}{c} \hat{\theta }\left(k+1\right)=\hat{\theta }\left(k\right)+K\left(k+1\right)\left[y\left(k+1\right)-\phi^{T}\left(k+1\right)\hat{\theta }\left(k\right)\right]\\ K\left(k+1\right)=P\left(k+1\right)\phi \left(k+1\right){\left[\lambda +\phi^{T}\left(k+1\right)P\left(k\right)\phi \left(k+1\right)\right]}^{-1}\\ P\left(k+1\right)=\frac{1}{\lambda }\left[I-K\left(k+1\right)\phi^{T}\left(k+1\right)\right]P\left(k\right) \end{array}\right.$$

2. Substituting $\hat{\theta }={\left[\phi^{T}\phi \right]}^{-1}\phi^{T}Y$, the parameter $a_{i}\left(i=1,2,...,5\right)$ can be obtained.

1. Use the inverse bilinear rule for the transfer function of Equation (3), let $z^{-1}=\left(1-\frac{\Delta T}{2}s\right)/\left(1+\frac{\Delta T}{2}s\right)$, then:

(10) $$G\left(s\right)=\frac{\frac{a_{3}-a_{4}+a_{5}}{1+a_{1}-a_{2}}s^{2}+\frac{4\left(a_{3}-a_{5}\right)}{\Delta T\left(1+a_{1}-a_{2}\right)}s+\frac{4\left(a_{3}-a_{4}+a_{5}\right)}{\Delta T^{2}\left(1+a_{1}-a_{2}\right)}}{s^{2}+\frac{4\left(1+a_{2}\right)}{\Delta T\left(1+a_{1}-a_{2}\right)}+\frac{4\left(1-a_{1}-a_{2}\right)}{\Delta T^{2}\left(1+a_{1}-a_{2}\right)}}$$

$R_{0}$, $R_{1}$, $R_{2}$, $C_{1}$, and $C_{2}$ can be obtained by comprehensively comparing the corresponding coefficients in steps 1 and 7. The identification results are shown in Table 1.

### 2.3. Model Validation

**Figure 3:**
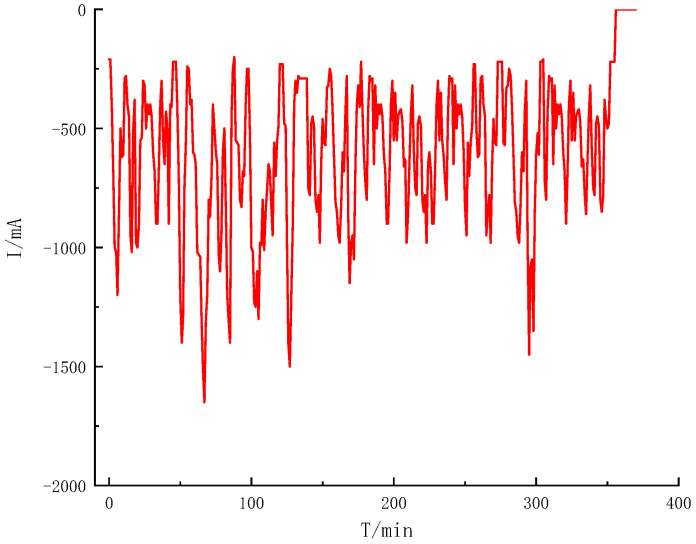
UDDS working current diagram.

**Figure 4:**
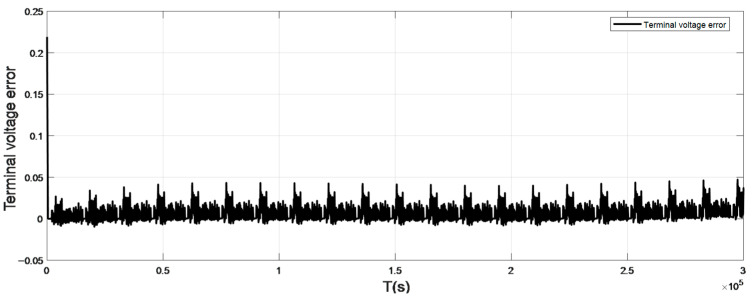
Terminal voltage error curve at 25°C.

**Figure 5:**
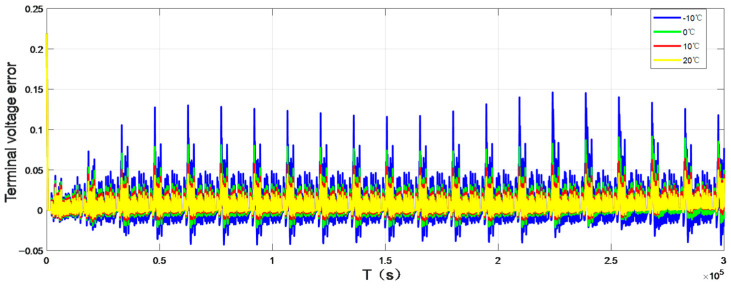
Terminal voltage error curve at low temperature.

**Figure 6:**
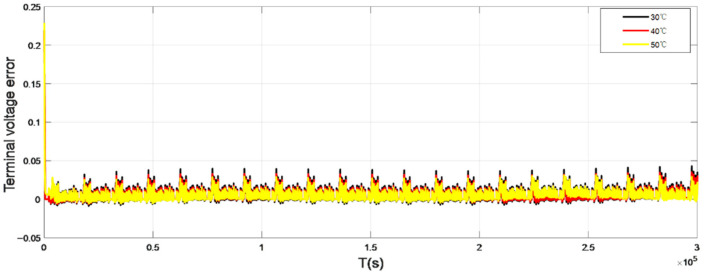
Terminal voltage error curve at high temperature.

To verify the accuracy of the model, this paper selected the UDDS (Urban dynameter Driving Schedule) working condition to verify, and the working current is shown in Figure 3. The terminal voltage error curve under normal atmospheric temperature 25 °C is shown in Figure 4. Figure 5 and Figure 6 show the terminal voltage error curves of UDDS operating at low (<25 °C) and high (>25 °C) temperatures.

The model input was defined as the working current and temperature. Figure 3 is the working current diagram in this test environment. The output is the terminal voltage value estimated by the model. The terminal voltage value can describe the polarization phenomenon of the battery and conform to the voltage characteristics of the battery. Therefore, the difference between the output terminal voltage of the model and the real terminal voltage can evaluate the accuracy of the power battery model. The UDDS working condition experiment was carried out on the power battery at different temperature nodes, and the following conclusions were drawn.

Compared with the working current waveform of UDDS, the terminal voltage error at the small current section (current ≤ 1A) is mostly maintained at ±100 mV, and the voltage error at the large current section (current > 1A) can reach 150–200 mV. It can be seen from Figure 5 that the terminal voltage error at −10 °C fluctuated greatly, and the terminal voltage error of 0 °C was greatly improved, which can maintain ±50 mV in the small current section, and the error range is 50–100 mV in the large current section. As shown in Figure 6, when the ambient temperature is more than 25 °C, the fluctuation range of battery model error can be basically kept within ±20 mV, and even the maximum error of terminal voltage in the large current section is about 50 mV. It can be seen that the overall error of the battery model is small and the DP equivalent circuit model has good adaptability.

## 3. SOC Estimation Based on Adaptive Sliding Mode Observer

The adaptive Sliding Mode Observer [31] can estimate the SOC of the lithium-ion battery in electric vehicles. Whether the initial value of SOC is known or not, this method can estimate the SOC with high accuracy and less computation only by using the measured current and voltage values. It can overcome the nonlinearity, external interference, and measurement noise of the battery model, which is suitable for complex operating conditions. To adapt to the application of the adaptive Sliding Mode Observer, the state space equation of the DP equivalent circuit model is established. Voltages $u_{1}$ and $u_{2}$ on $R_{1}$ and $R_{2}$ are chosen as the two state quantities of the system, so $x_{1}=u_{1}$, $x_{2}=u_{2}$, $x_{3}=soc$ and input u are currents, which are positive when discharging and negative when charging. By writing the corresponding relationship between voltage and current according to the circuit principle, the state-space model of the cell can be obtained as shown in Equation (11):

(11) $$\left\{\begin{array}{l} \left[\begin{array}{c} {\dot{x}}_{1}\\ {\dot{x}}_{2}\\ {\dot{x}}_{3} \end{array}\right]=\left[\begin{array}{ccc} -\frac{1}{R_{1}C_{1}} & 0 & 0\\ 0 & -\frac{1}{R_{2}C_{2}} & 0\\ 0 & 0 & 0 \end{array}\right]\left[\begin{array}{c} x_{1}\\ x_{2}\\ x_{3} \end{array}\right]+\left[\begin{array}{c} \frac{1}{C_{1}}\\ \frac{1}{C_{2}}\\ -\frac{\eta }{Q_{N}} \end{array}\right]u\\ y=f\left(x_{3}\right)-x_{1}-x_{2}+R_{0}u \end{array}\right.$$

In the formula, η is the discharge efficiency, ideally 1, $f\left(x_{3}\right)$ is the relationship between the open circuit voltage and SOC of the battery.

According to the state space equation of the DP equivalent circuit model, an adaptive Sliding Mode Observer is designed. Suppose that $\dot{\hat{x}}\left(i=1,2,3\right)$ is the state of the estimated system based on the SMO and $\hat{y}$ is the output of the estimated system. The structure of the design estimator is shown in Equation (12):

(12) $$\left\{\begin{array}{c} \dot{\hat{x}}=A\hat{x}+Bu-Le_{y}-P{\mathrm{sgn}}_{a}\left(e_{y}\right)\\ \hat{y}={\hat{x}}_{1}+{\hat{x}}_{2}+f\left({\hat{x}}_{3}\right)+Du \end{array}\right.$$

$e_{y}=y-\hat{y}$ is the systematic error of SOC estimation, $L={\left[\begin{array}{ccc} l_{1} & l_{2} & l_{3} \end{array}\right]}^{T}$ and $P={\left[\begin{array}{ccc} \rho_{1} & \rho_{2} & \rho_{3} \end{array}\right]}^{T}$ are, respectively, the Lomberg feedback gain and sliding-mode variable structure feedback gain.

Substitute class symbolic function ${\mathrm{sgn}}_{a}\left(e_{y}\right)=\frac{e_{y}}{\left|\left(e_{y}\right)\right|+\lambda }$ into Equation (12) and define $s_{i}=l_{i}+\frac{\rho_{i}}{\left|e_{y}\right|+\lambda },\left(i=1,2,3\right)$, $S=\left[s_{1},s_{2},s_{3}\right]$ is the observer gain matrix. According to Equations (11) and (12), the observer can be written as:

(13) $$\left\{\begin{array}{l} {\dot{\hat{x}}}_{1}=-\frac{1}{R_{1}C_{1}}{\hat{x}}_{1}+\frac{1}{C_{1}}u-l_{1}e_{y}-\rho_{1}{\mathrm{sgn}}_{a}\left(e_{y}\right)=-\frac{1}{R_{1}C_{1}}{\hat{x}}_{1}+\frac{1}{C_{1}}u-s_{1}e_{y}\\ {\dot{\hat{x}}}_{2}=-\frac{1}{R_{2}C_{2}}{\hat{x}}_{2}+\frac{1}{C_{2}}u-l_{2}e_{y}-\rho_{2}{\mathrm{sgn}}_{a}\left(e_{y}\right)=-\frac{1}{R_{2}C_{2}}{\hat{x}}_{2}+\frac{1}{C_{2}}u-s_{2}e_{y}\\ {\dot{\hat{x}}}_{3}=-\frac{\eta }{Q_{N}}u-l_{3}e_{y}-\rho_{3}{\mathrm{sgn}}_{a}\left(e_{y}\right)=-\frac{\eta }{Q_{N}}u-s_{3}e_{y} \end{array}\right.$$

(14) $$l_{i}<s_{i}=l_{i}+\frac{\rho_{i}}{\left|e_{y}\right|+\lambda }\leq l_{i}+\frac{\rho_{i}}{\lambda }$$

To analyze the convergence of the observer, the state estimation error is defined as ${\bar{x}}_{i}=x_{i}-{\hat{x}}_{i}\left(i=1,2,3\right)$, then the estimation error system can be written as:

(15) $$\left\{\begin{array}{l} {\dot{\bar{x}}}_{1}=-\frac{1}{R_{1}C_{1}}{\bar{x}}_{1}+s_{1}e_{y}\\ {\dot{\bar{x}}}_{2}=-\frac{1}{R_{2}C_{2}}{\bar{x}}_{2}+s_{2}e_{y}\\ \dot{\bar{x}}=s_{3}e_{y} \end{array}\right.$$

According to Lagrange’s median theorem, the output error equation is written as:

(16) $$e_{y}=y-\hat{y}={\bar{x}}_{1}+{\bar{x}}_{2}+\left(f^{\prime }\left(\xi \right)\right){\bar{x}}_{3}\;\xi \in \left[x_{3},{\bar{x}}_{3}\right]$$

Choose Lyapunov function:

(17) $$V=\frac{1}{2}{\bar{x}}_{1}^{2}+\frac{1}{2}{\bar{x}}_{2}^{2}+\frac{1}{2}{\bar{x}}_{3}^{2}$$

Then the sufficient condition for the stability of the observer is as follows:

(18) $$\dot{V}={\bar{x}}_{1}{\dot{\bar{x}}}_{1}+{\bar{x}}_{2}{\dot{\bar{x}}}_{2}+{\bar{x}}_{3}{\dot{\bar{x}}}_{3}<0$$

Equation (19) can be obtained by Equations (16) and (18):

(19) $$\begin{array}{l} \dot{V}=\left(a_{11}+s_{1}\right){\bar{x}}_{1}^{2}+\left(a_{22}+s_{2}\right){\bar{x}}_{2}^{2}+s_{3}f^{\prime }\left(\xi \right){\bar{x}}_{3}^{2}\\ +\left(s_{1}+s_{2}\right){\bar{x}}_{1}{\bar{x}}_{2}+\left(s_{1}f^{\prime }\left(\xi \right)+s_{3}\right){\bar{x}}_{1}{\bar{x}}_{3}+\left(s_{2}f^{\prime }\left(\xi \right)+s_{3}\right){\bar{x}}_{2}{\bar{x}}_{3} \end{array}$$

The sufficient condition for the stability of the above formula is that the following matrix H is positive definite:

(20) $$H=\left[\begin{array}{ccc} -s_{1}-a_{11} & -\frac{s_{1}+s_{2}}{2} & -\frac{s_{1}f^{\prime }\left(\xi \right)+s_{3}}{2}\\ -\frac{s_{1}+s_{2}}{2} & -s_{2}-a_{22} & -\frac{s_{2}f^{\prime }\left(\xi \right)+s_{3}}{2}\\ -\frac{s_{1}f^{\prime }\left(\xi \right)+s_{3}}{2} & -\frac{s_{2}f^{\prime }\left(\xi \right)+s_{3}}{2} & -s_{3}f^{\prime }\left(\xi \right) \end{array}\right]$$

Let $m_{1}=\frac{1}{R_{1}C_{1}}$, $m_{2}=\frac{1}{R_{2}C_{2}}$, and the sufficient condition for the convergence of the observer is as follows.

(21) $$\left\{\begin{array}{c} s_{1}>-m_{1}\\ m_{1}+s_{1}-2\sqrt{\left(m_{1}+m_{2}\right)\left(s_{1}+m_{1}\right)}<s_{2}<m_{1}+s_{1}+2\sqrt{\left(m_{1}+m_{2}\right)\left(s_{1}+m_{1}\right)}\\ \begin{array}{l} \frac{{f^{\prime }}_{oc}\left(\xi \right)}{\left(m_{1}+m_{2}\right)}\left(-\left(m_{1}s_{1}+m_{2}s_{1}+2m_{1}m_{2}\right)-g_{1}\left(s_{1},s_{2}\right)\right)<s_{3}<\frac{{f^{\prime }}_{oc}\left(\xi \right)}{\left(m_{1}+m_{2}\right)}\\ \left(-\left(m_{1}s_{1}+m_{2}s_{1}+2m_{1}m_{2}\right)+g_{1}\left(s_{1},s_{2}\right)\right) \end{array} \end{array}\right.$$

Since SOC varies from 0 to 1, it can be determined that $f^{\prime }\left(\xi \right)>0$ is always established, so $f_{oc}\left(x\right)$ is a monotonically increasing function, and its derivative is bounded, that is, $0<f_{oc}^{\prime }\left(0\right)\leq f_{oc}^{\prime }\left(1\right)$.

Set $f_{oc}^{\prime }\left(\xi \right)$, replace with the boundary value, and scale the third inequality in Equation (21) to get Equation (22):

(22) $$\begin{array}{l} \frac{f^{\prime }\left(1\right)}{\left(m_{1}+m_{2}\right)}\left(-\left(m_{1}s_{2}+m_{2}s_{1}+2m_{1}m_{2}\right)-g_{1}\left(s_{1},s_{2}\right)\right)<s_{3}\\ <\frac{f^{\prime }\left(0\right)}{\left(m_{1}+m_{2}\right)}\left(-\left(m_{1}s_{2}+m_{2}s_{1}+2m_{1}m_{2}\right)+g_{1}\left(s_{1},s_{2}\right)\right) \end{array}$$

In this way, the sufficient conditions for the state gain matrix parameters to converge are obtained. Combined with the identified model parameters, the parameter function expressions $m_{1}=\frac{1}{R_{1}\left(T\right)C_{1}\left(T\right)}$ and $m_{2}=\frac{1}{R_{2}\left(T\right)C_{2}\left(T\right)}$ can be derived. By substituting these parameter forms into Equations (20) and (21), the feedback gain of the Sliding Mode Observer can be obtained, i.e., $m_{1}\in \left[0.009,0.024\right]$,$\;m_{2}\in \left[0.0004,0.0016\right]$.

The range of $s_{i}$ is obtained using the calculation of $l_{i}$ and $\rho_{i}$. Set $l_{1}=0.28$ and $\rho_{1}=0.03$ as initial values to satisfy the first condition in Equation (21). Where λ=0.1, the range of $s_{i}$ can be obtained as:

(23) $$l_{2}>0.062,\;0.28=l_{1}<s_{1}<l_{1}+\frac{\rho_{1}}{\lambda }=0.31$$

Substituting the range of $s_{1}$ into the second inequality of Equation (21), the range of $s_{2}$. $l_{2}>0.062$ can be solved and $l_{2}=0.8$ can be selected. From $0<\rho_{2}<0.083$, $l_{3}>-1.12$, $0<\rho_{3}<0.086$, $\rho_{2}=0.21$, $l_{3}=-0.186$, and $\rho_{3}=0.032\;$ can be chosen. The results of feedback gain are shown in Table 2.

**Figure 7:**
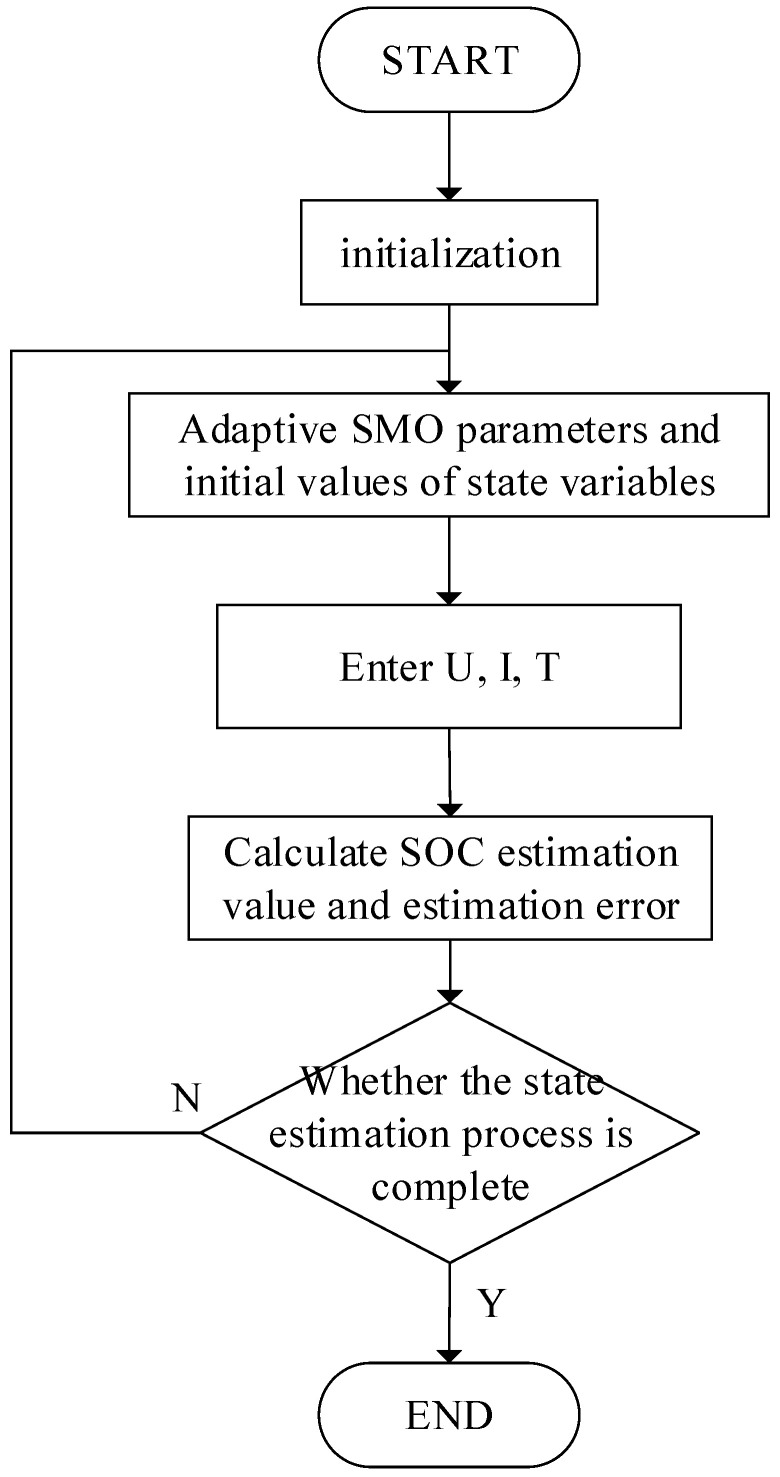
SOC estimation process.

Each sampling point is adjusted automatically to obtain the gain under the condition of the model parameter value by setting the observer gain parameters adaptively, so that the state feedback gain of the SMO satisfies all above inequalities. Therefore, the design of an adaptive Sliding Mode Observer for SOC estimation is completed. The SOC estimation process based on adaptive SMO is shown in Figure 7.

## 4. Experiments and Result Analysis

To verify the SOC estimation algorithm based on adaptive SMO, the experiments of the constant current discharge condition and UDDS condition under three different ambient temperatures of low temperature (0 °C), normal temperature (25 °C), and high temperature (50 °C) are designed. The initial value of the SOC under the constant current pulse discharge condition and UDDS condition is set to 0.8 in the simulation, while the real SOC starting point of the two conditions is 1. In the discharge process, the SOC measured by the IT8500 discharge instrument is taken as the measured value and compared with the battery SOC estimated value obtained by the estimation algorithm in this paper to verify the effectiveness of the algorithm.

### 4.1. Discharge Experiment Verification and Analysis at Low Temperature

**Figure 8:**
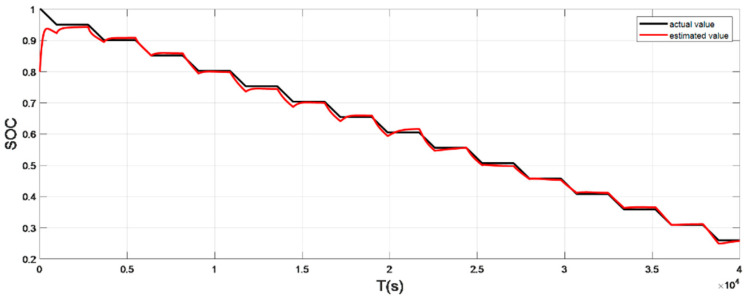
Comparison of the SOC real value and estimated value under constant current discharge (low temperature: 0 °C).

**Figure 9:**
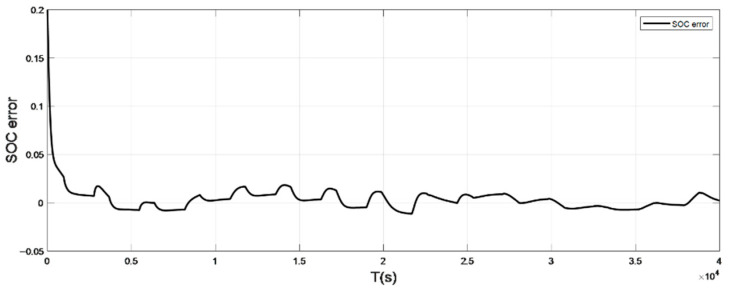
The SOC estimation error under constant current discharge (low temperature: 0 °C).

**Figure 10:**
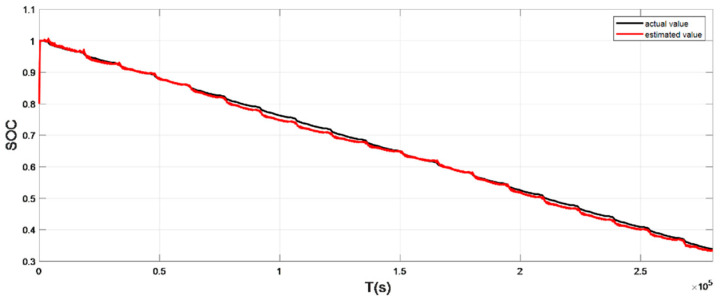
Comparison of the SOC real value and estimated value under UDDS (low temperature: 0 °C).

**Figure 11:**
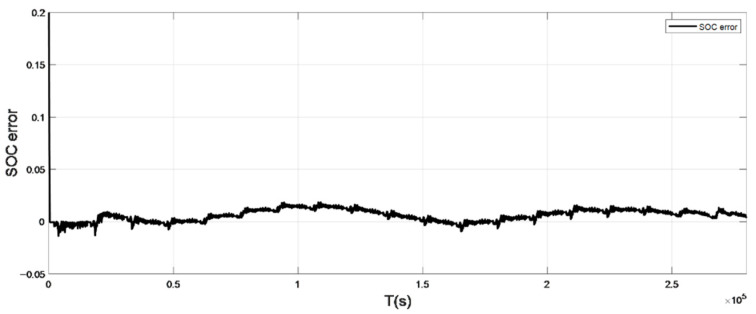
The SOC estimation error under UDDS (low temperature: 0 °C).

The input ambient temperature is 0 °C, and the initial SOC value is set to 0.8 in the constant current pulse discharge condition and UDDS condition in the SOC estimation using the adaptive Sliding Mode Observer, and the SOC starting point in the discharge experiment to obtain the real value is 1. The SOC estimation results are shown in Figure 8, Figure 9, Figure 10 and Figure 11.

It can be seen from the graph that the real and estimated SOC values converge in a trapezoidal shape under the condition of constant current pulse discharge. The UDDS working condition is the process of continuously discharging the battery, which is in dynamic change. Although the initial SOC values set in the estimation strategy are different, the estimated value with a large initial error can still converge to the real value in 127 s under the UDDS condition, and the overall deviation is kept within 2%.

Simulation results show that even if the initial SOC set in the estimation strategy is different, it can still converge the estimated value with a larger initial error to the real value in a short time period and keep the overall error within 2%.

### 4.2. Discharge Experiment Verification and Analysis at Normal Temperature

**Figure 12:**
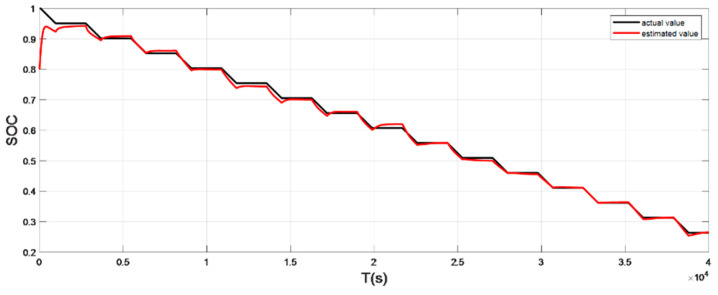
Comparison of the SOC real value and estimated value under constant current discharge (normal temperature: 25 °C).

**Figure 13:**
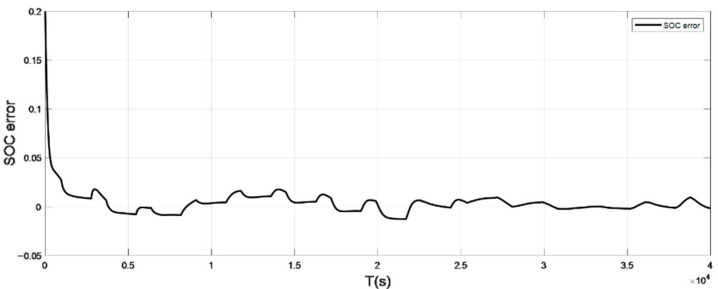
The SOC estimation error under constant current discharge (normal temperature: 25 °C).

**Figure 14:**
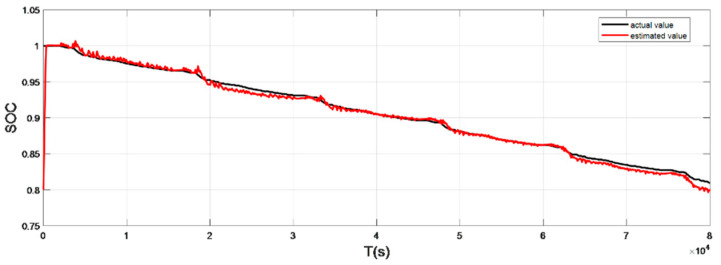
Comparison of the SOC real value and estimated value under UDDS (normal temperature: 25 °C).

**Figure 15:**
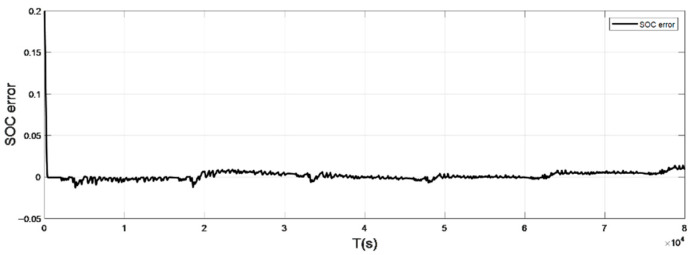
The SOC estimation error under UDDS (normal temperature: 25 °C).

The input ambient temperature is 25 °C, and the initial SOC value is set to 0.8 in the constant current pulse discharge condition and UDDS condition in the SOC estimation using the adaptive Sliding Mode Observer, and the SOC starting point in the discharge experiment to obtain the real value is 1, the SOC estimation results are shown in Figure 12, Figure 13, Figure 14 and Figure 15.

It can be seen from the simulation results that the SOC estimation strategy at room temperature has faster convergence speed and higher accuracy, and the overall error is within 1%. Compared with the SOC estimation result at low temperature, the error of the SOC simulation results at room temperature is smaller. The main reason is that the internal chemical reaction of the battery at room temperature is in a stable state, so the estimated value at room temperature is very close to the real value, and the error between the two is smaller.

### 4.3. Discharge Experiment Verification and Analysis at High Temperature

**Figure 16:**
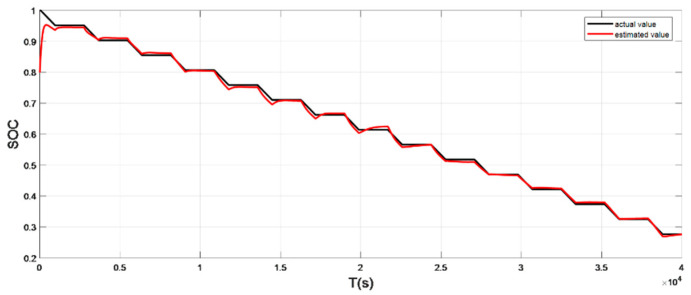
Comparison of the SOC real value and estimated value under constant current discharge (high temperature: 50 °C).

**Figure 17:**
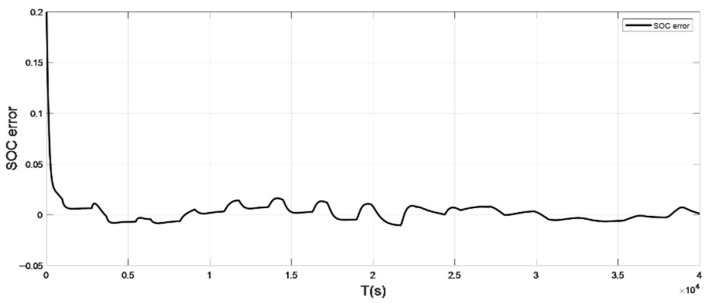
The SOC estimation error under constant current discharge (high temperature: 50 °C).

**Figure 18:**
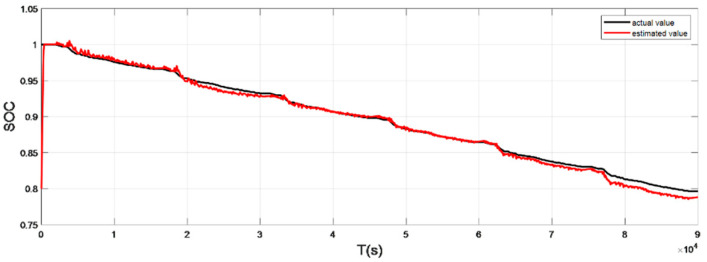
Comparison of the SOC real value and estimated value under UDDS (high temperature: 50 °C).

**Figure 19:**
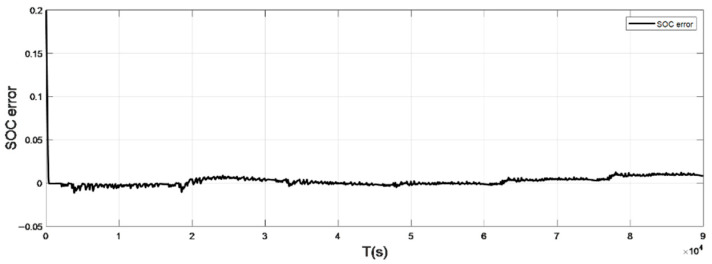
The SOC estimation error under UDDS (high temperature: 50 °C).

The input ambient temperature is 50 °C and the initial SOC value is set to 0.8 in the constant current pulse discharge condition and UDDS condition in the SOC estimation using the adaptive Sliding Mode Observer, while the SOC starting point in the discharge experiment to obtain the real value is 1. The real SOC starting point of the two working conditions is 1, and the SOC estimation results are shown in Figure 16, Figure 17, Figure 18 and Figure 19.

It can be seen from the simulation results that the SOC estimation strategy proposed in this paper has good adaptability at high temperature, the overall error is within 2%, and the estimated value can still converge to the real value in 146 s.

The estimation error results of three different temperatures (low temperature 0 °C, room temperature 25 °C, and high temperature 50 °C) under constant current discharge and UDDS conditions are shown in Table 3. The adaptive Sliding Mode Observer estimation SOC mentioned in this paper can converge quickly in different temperatures, and the convergence times are all less than 200 s. When the initial SOC value is uncertain or even has a large deviation from the actual value, the adaptive Sliding Mode Observer can make the estimated value converge to the actual value stably, and the estimation effect of the battery is well under different initial charging states. The estimation method based on adaptive Sliding Mode Observer has strong robustness and tracking ability to state variables and is suitable for constant flow and complex road conditions.

## 5. Conclusions

In this paper, the second-order DP equivalent circuit model of a lithium-ion battery was established, and the parameters of the DP model were identified using a discharge experiment and least square method with a forgetting factor. A SOC estimation algorithm based on adaptive Sliding Mode Observer was proposed and verified by discharge experiments at different ambient temperatures. The experimental results show that the SOC estimation error of the algorithm is less than 2% at low and high temperatures, and the convergence speed is 127 and 146 s, respectively, under UDDS conditions. The SOC estimation error is less than 1 % at the normal temperature, and the convergence speed is 181 s. The algorithm has high accuracy, robustness, and the requirements of engineering practice.

## Figures and Tables

**Table 1:** Parameter identification results of the FFLS method.

	$R_{0}\left(\Omega \right)$	$R_{1}\left(\Omega \right)$	$R_{2}\left(\Omega \right)$	$C_{1}\left(F\right)$	$C_{2}\left(F\right)$
−10 °C	0.1111	0.1271	0.3659	789.1385	7080.9
0 °C	0.0664	0.0645	0.1224	1207.0	9674.2
10 °C	0.0499	0.0395	0.0455	1235.9	13,249.0
20 °C	0.0411	0.0310	0.0251	1546.7	23,542.0
25 °C	0.0377	0.0291	0.0217	1674.1	24,047.0
30 °C	0.0385	0.0265	0.0211	1711.9	31,205.0
40 °C	0.0314	0.0206	0.0137	2083.0	49,088.0
50 °C	0.0288	0.0174	0.0106	2347.3	58,764.0

**Table 2:** Feedback gain table of the Sliding Mode Observer.

**Parameter**	$l_{1}$	$l_{2}$	$l_{3}$	$\rho_{1}$	$\rho_{2}$	$\rho_{3}$
**Parameter value**	0.27	0.82	−0.175	0.04	0.22	0.034

**Table 3:** Estimation error table.

Conditions	Errors	Temperatures		
		0 °C	25 °C	50 °C
Constant current discharge condition	Maximum error	2.26%	1.16%	2.23%
	Average error	1.14%	0.83%	1.29%
UDDS condition	Maximum error	2.19%	1.98%	1.86%
	Average error	1.28%	1.35%	1.37%

## Data Availability

Not applicable.
